# Gelatinizing oil in water and its removal via bacteria inhabiting the gels

**DOI:** 10.1038/s41598-017-14296-x

**Published:** 2017-10-25

**Authors:** Samir S. A. Radwan, Dina M. Al-Mailem, Mayada K. Kansour

**Affiliations:** Microbiology Program, Department of Biological Sciences, Faculty of Science, Kuwait University, P O Box 5969, Safat, 13060 Kuwait

## Abstract

When crude oil samples were shaken (200 rpm) in seawater samples from the Arabian Gulf at 30 °C, usually oil-gels were produced spontaneously leaving the water quite clear. The gelators could probably be based on cholesteryl derivatives. Microscopic examination of the established gels revealed nanofibrellar structures similar to those described by earlier workers for artificially synthesized gelators. Communities of bacteria including prosthetic and stalked members as well as oil-degrading bacteria were recorded in such gels. Chemical analysis revealed that 88.5% of the oil entrapped by gelation was biodegraded after 40 days at 30 °C. Individual bacterial species isolated from the oil-gels biodegraded in batch cultures between 17.8 and 33.3% of the oil added at time zero in 12 days at 30 °C. Gelation is a promising approach, not only for clean, physical removal of oil spilled in aquatic habitats, as so far suggested, but also in its effective microbiological biodegradation, as the current study revealed.

## Introduction

In the last few decades, interest of chemists, particularly physical chemists has been increasingly focused on the socalled organic gelators and their technical applications. These are small, structurally diverse organic molecules capable of gelatinizing organic liquids by creating supramolecular networks by self-assembly, which entrap the organic-solvent molecules^1,2^. Of special interest are the self-assembled viscoelastic gels of organic solvents and water^3^ that have promising, high-tech application potential in diverse fields including environmental remediation^4^. Gelators known so far, range in chemical composition from simple alkanes^5^ to complex ether derivatives^6^. Forces mediating the self-assembling process comprise non-covalent interactions, electrostatic stacking, hydrogen bonding and others^2^. Gelators reported in the literature so far, have been artificially synthesized and gelated in response to stimuli like heat, pH, mechanical agitation, electromagnetic fields and others^7^. Although studies in the literature on gelators have put major emphasis on their physicochemical properties, questions in this area of research regarding the mechanism(s) of their construction are still much more than answers.

Of particular relevance to the current work are the relatively few publications on gelation of crude oil from waters^8,9^. Those studies provide information that may be implemented in suggesting technologies for physical removal of oil spilled in the aquatic environment. Within this context, other materials currently in use for physicochemical oil remediation are sorbents^10,11^, solidifiers^12^ and dispersants^13^. The use of such materials is however, frequently unsafe, and /or not eco-friendly^14^.

An early publication on the chemistry of an oil-gelator (from water) was that based on an alanine amphiphile^9^. Subsequent reports were on other oil-gelators based on amino acids^15^, peptides bearing side-chain azobenzene moieties^16^, peptides of synthetic β-amino acids^17^ and sugar-based gelators^18^. There are also oil-gelators based on cholesteryl derivatives^19,20^. In such studies, gelation could only happen after heating-cooling cycles, a factor which would be an obstacle in field application. Therefore, biotechnologists commonly recommend the design of gelators effective at moderate (room) temperatures.

As mentioned above the subject of gelators has so far received the interest of mainly chemists and physical-chemists. In this contribution, we show that this subject is equally interesting from the microbiological view point. We provide experimental evidence that naturally gelatinized crude oil harbors unique microbial communities quite effective in oil biodegradation. Thus, oil-gelators do not only remove spilled oil by mere oil entrapping, but also by bringing closely together oil and hydrocarbonoclastic microorganisms that mineralize this pollutant.

## Results

### The oil-gelation process

Through 25-year-research on bioremediation of oil-polluted seawater from the Arabian Gulf, our group observed the spontaneous oil-gelation process quite frequently. This phenomenon was recorded in batch experiments in which seawater samples in conical flasks were mixed with oil and shaken electrically, 100–200 rpm, at 30 °C for several days. We assumed that those gels could be nothing but extracellular polymers released by the cells to facilitate oil uptake and biodegradation by the microorganisms^21,22^.

To study the specific conditions under which this natural oil-gelation may occur, we collected coastal seawater samples from 4 stations along the 100 km Kuwaiti coast of the Arabian Gulf. Those stations, which were about 30 km apart, were from north to south, Subiya, Kuwait Towers, Fintas and Khiran. The coastal samples were collected in sterile glass containers and transported to the laboratory for processing.

**Figure 1:**
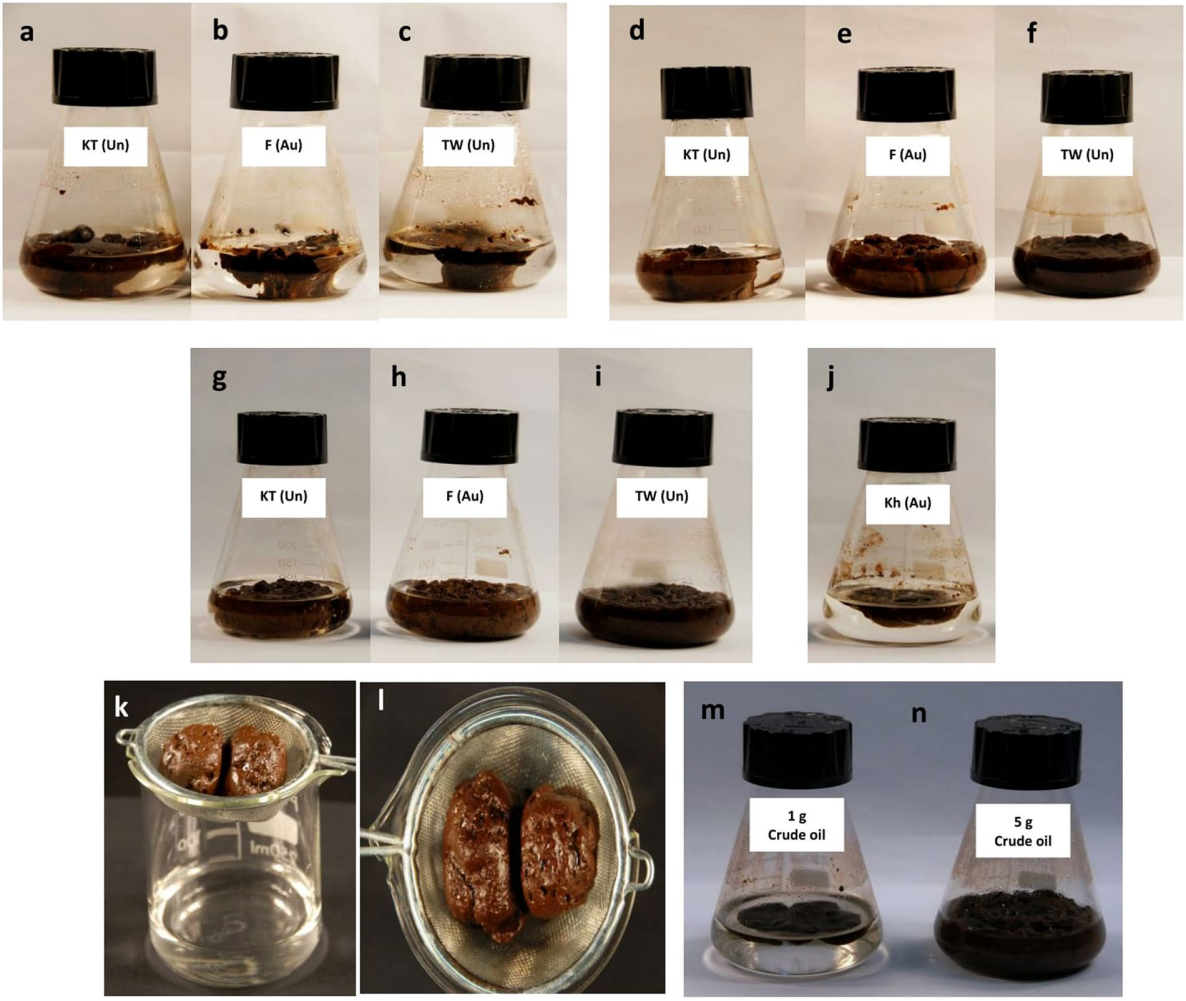
Natural oil-gelation in shaken (200 rpm) water samples. (**a,b,c**) Gels established after 4 days in Kuwait Towers (KT), Fintas (F) and tap water (TW) samples, respectively. (**d,e,f**) Gels established in the same samples after 40 days. (**g**,**h**,**i**) Gels established in the same samples after 4 months. Those results were obtained when previously autoclaved (Au) and unautoclaved (Un) water samples were used. (**j**) Gel established in Khiran (Kh) water, but first after 40 days. (**k**,**l)** Gels established after 40 days in Kuwait Towers water, note how clear the seawater was left after removing the gel. (**m**,**n**) Gels established after only 24 hours in Kuwait Towers waters inoculated with 1 g pieces of previously established gels.

Shaking (200 rpm) crude oil (3 g) in 100 ml of coastal water samples from the different sites at 30 °C for up to 4 months (mostly a few days was enough, see Fig. 1) led to successful, spontaneous oil gelation in many, but not in all cases. Consistent was that successful gelation strictly dependended on effective shaking. There was no gelation at all when parallel replicate samples were stationary incubated at 30 °C. Mechanical agitation was also reported by earlier investigators working on artificially synthesized oil gelators to be among the major factors effective in gelation^7^. In our experiments, no gelators were deliberately added and the first sign of gelation was after about 4 days when the crude oil in about one half of the Kuwait Towers and Fintas water samples, but not of the Khiran and Subiya samples, turned into semi-solid masses (Fig. 1a,b). This was true although the environmental parameters in all sites were not dramatically different. Interestingly, similar semi-solid oil masses also started to appear in similarly treated tap water samples (Fig. 1c). This may imply that the natural oil gelating material was associated mainly with the oil, not with the water samples. The gelation frequencies were similar when fresh and previously autoclaved water samples were used. This excludes any role for microorganisms in the gelation process. Our experiments also showed that the oil-gelation frequency was higher with heavy than with light crude oil samples. The gelation processes became “mature” after 40 days (Fig. 1d–f), and perfectly solid gels were structured after 4 months (Fig. 1g–i). Such gels also formed sometimes in the Khiran (Fig. 1j), but not at all in the Subiya water samples. After 40 days (and also after 4 months), oil-gels that developed naturally could be cleanly filtered from the clear seawater using conventional metal sieves (Fig. 1k,l). In another experiment, we mixed separately 1 g and 5 g heavy crude oil with 100 ml seawater samples from the Kuwait Towers site and inoculated the mixtures with 1 g portions of already established oil gels. After shaking, 200 rpm, at 30 °C, the gelation process was surprisingly perfect after only 24 hours (Fig. 1m,n), i.e. it was enhanced.

The above observations imply that there are natural oil-gelators in crude oil samples which have the potential for self assembly and gelating oil in water at 30 °C. Already established gels are capable of enhancing the gelation process when small pieces of those gels are shaken with the water-oil mixtures, probably by acting as “starters” in the self-assembly process. Within this context, most, if not all of the oil-gelators studied earlier had the limitation that they were produced synthetically and failed to produce intact, solid gels at moderate (room) temperatures^7^. Those features would obviously limit their potential application in remediation of environmental oil spills^23^. On the other hand, our results like those of earlier investigators still raise more questions than answers regarding the nature of such “natural” gelators, a subject which we will still elaborate on below.

### Chemical aspects of the natural oil-gelators

As already mentioned, oil-gelators studied so far, were artificially synthesized materials based on organic chemicals such as amino acids^15^ and peptides^17^, carbohydrates^18^ and cholesteryl derivatives^19,20^. Elemental analysis of our four month oil-gels, in which most of the oil had already been mineralized (see below), revealed that they consisted of 12.7% carbon, 6.9% hydrogen, 2.3% nitrogen and 1.0% sulfur. In addition, those gels contained 12.1 mg g^−1^ of calcium ions and 1.0 mg g^−1^ of sodium ions. Although nitrogen and sulfur were present, those values are quite dissimilar to the elemental analytical values expected for amino acids or peptides. We subjected our oil-gels to acid (1 N H_2_SO_4_) and alkaline (1 N NaOH) hydrolysis and the subsequent paper (PC) and thin layer (TLC) chromatographic analysis (Material and methods) showed that the hydrolysates contained no amino acids. Instrumental analysis by liquid chromatography combined with mass spectrometry (LC/MS) also failed to detect any peptides or amino acids in the gelators. Thus, we excluded the possibility that the natural gelators that gelatinized crude oil could be amino acid- or peptide-based. Similarly, PC and TLC of the gel hydrolysates failed to detect any monosaccharides, thus also excluding that the natural gelators producing those oil-gels could be sugar-based.

**Figure 2:**
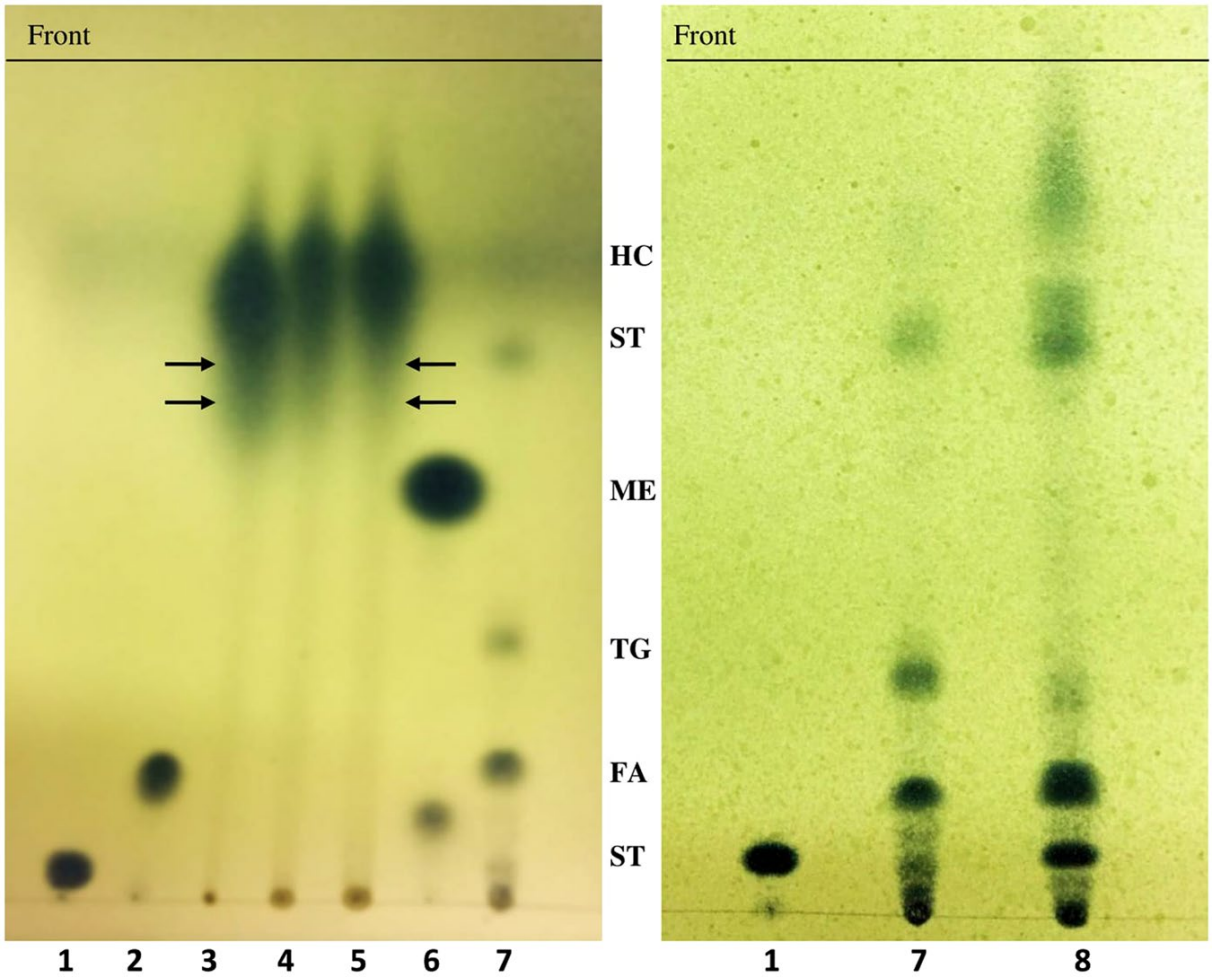
TLC-Analysis of lipid classes in total lipids. Sorbent: Silica Gel G. Running solvent: Hexane, diethyl ether, acetic acid, 85:15:1, by vol. Visualization: Phosphomolybdic acid and 50% H_2_SO_4_, at 120 °C. 1, Standard cholesterol; 2, standard oleic acid; 3, crude oil extract; 4&5, oil-gel extracts; 6, standard methyl oleate; 7, mixture of the standard samples; 8, fish extract. Spot identities: ST, sterols; FA, fatty acids; TG, triglycerides; ME, methyl esters of fatty acids; ST, steryl esters; HC, hydrocarbons.

The TLC analysis of total lipids from our 4 months old oil-gels provided some tentative experimental evidence supporting the assumption that the natural gelators involved in oil-gelation could be based on cholesteryl derivatives like those synthesized recently by chemists^23,24^. The TLC plates show that lipid extracts from crude oil and oil-gel exhibited in addition to hydrocarbons, TLC spots similar in their chromatographic behavior to that of standard cholesteryl oleate (Fig. 2, arrows). However, it is well known that most prokaryotes, in contrast to eukaryotes, lack sterols in their total lipids, and that cholesterol is a typical animal sterol. Therefore, cholesteryl derivatives of which our natural gelators could presumably had been produced should had their origin in aquatic lower and higher animals, e.g. fish. The coanalyzed total lipids from the fish, *Nemipterus randalli*, inhabiting the Arabian Gulf, contained sterols and steryl esters (Fig. 2). Earlier researchers synthesized cholesterol-based and dimeric cholesterol based type compounds capable of gelating organic solvents in water at room temperature^23^. Interestingly, those artificially synthesized gelators, like our gelators, also contained nitrogen and sulfur atoms. In the course of biosynthesis of our natural gelators, nitrogen and sulfur present in the resin fraction of oil may have been used. Our gelators could thus actually be related to the class of the cholesterol-based derivatives, yet more analysis is still needed to confirm that experimentally.

### Microscopic observations

**Figure 3:**
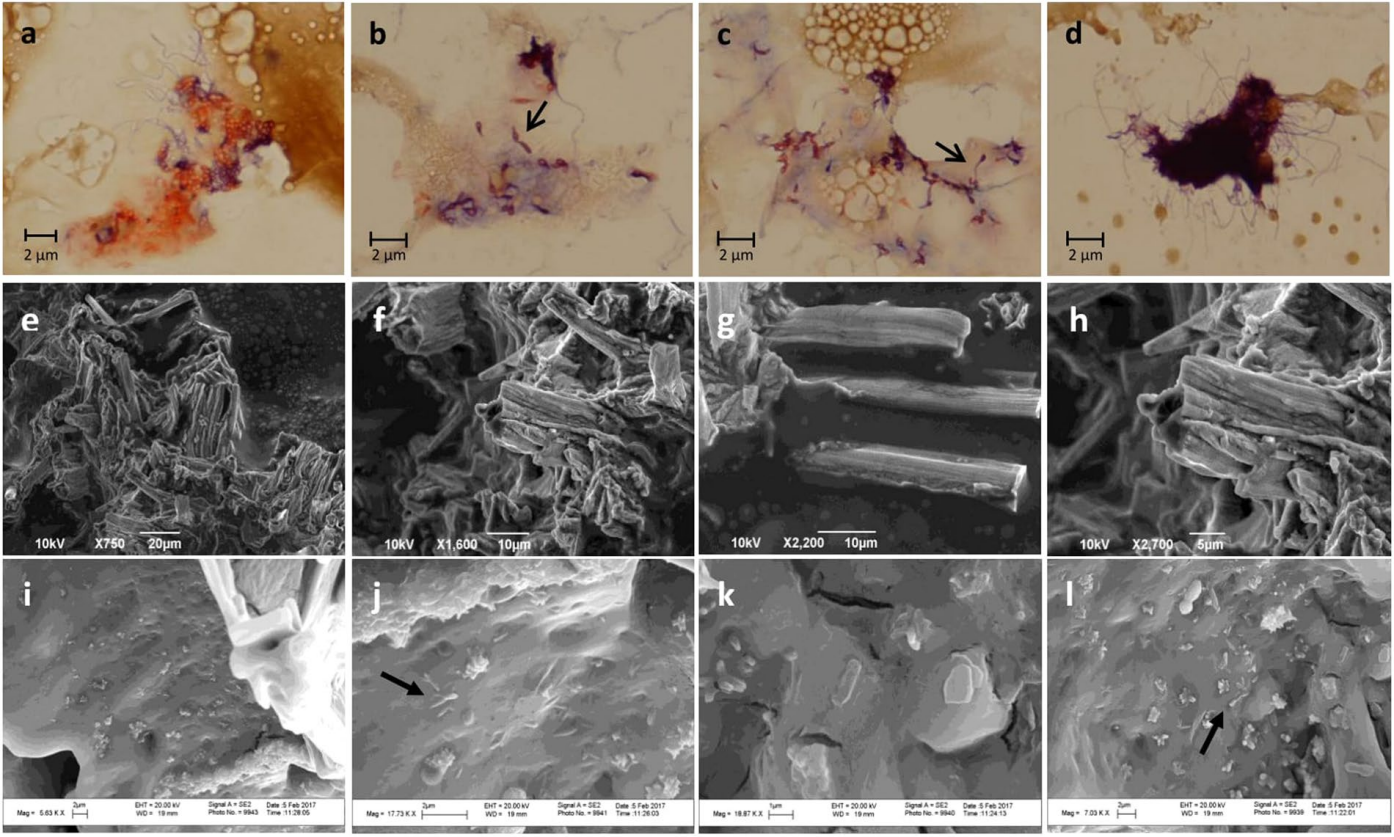
Light (**a**–**d**) and scanning electron (**e**–**l**) micrographs of 40 day oil-gel established in Fintas seawater sample. The light micrographs revealed the nanofibrillary structures (**a**,**d**) and the prosthetic (**b**, arrow) and stalked (**c**, arrow) bacteria. The prosthetic bacteria belonging to genus *Hyphomonas* showed the main bodies of the reproductive cells as well as the prosthecae and progenies. The stalked bacterial cells belonging to the genus *Maricaulis* had relatively long stalks. The scanning electron microscopic images showed the formation of nanofibrillar structures both in the previously autoclaved and unautoclaved samples and reveal bacterial cells in the unautoclaved (**i**–**l**) but not in the previously autoclaved (**e**–**h**) water samples.

In the phase-selective gelation process, gelators produce gels from oil-water mixture^9^. Such gelators are reportedly, self-assembled into nanofibrillar structures by noncovalent interactions^23,25^. We examined our 4 month gels by light- and electron-microscopy. Light-microscopy revealed extensive nanofibrillar structures (Fig. 3a–d) similar in morphology to those described by earlier investigators for synthetic gelators^16,23,26^. The scanning electron micrographs of gels prepared aseptically (using previously autoclaved seawater) (Fig. 3e–h) were also quite similar in morphology to those described by the same earlier investigators^16,23,26^. While the electron micrographs of those aseptic gels were free from bacteria, as should be expected, nonsterile gels (prepared using fresh, unautoclaved water) in contrast, appeared in light micrographs (Fig. 3b,c) as well as in scanning electron micrographs (Fig. 3j–l) colonized with bacterial cells. Many of such cells were those of prosthetic (Fig. 3b,c, arrow) and stalked (Fig. 3j,l, arrow) bacteria, whose identities are dealt with below.

### Bacterial communities in established oil-gels

In this experiment, our oil-gels and crude oil samples were analyzed for their bacterial communities using culture-dependent and culture-independent methods. Those gels had been established using fresh (unautoclaved) water samples from the Fintas site.

**Figure 4:**
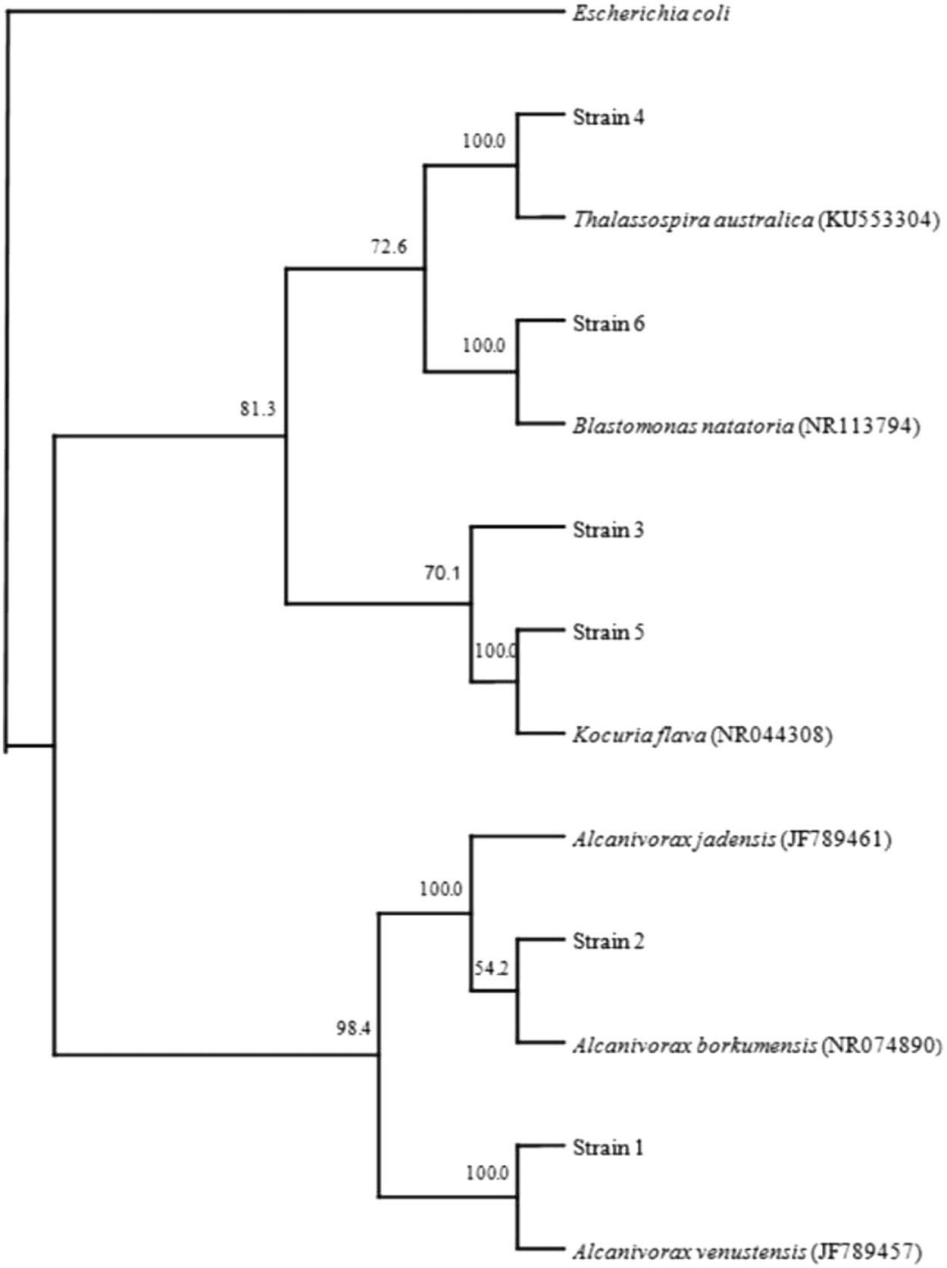
Culture-dependent analysis of bacterial communities associated with established oil-gels. Phylogenetic tree of 16S rRNA genes of the isolated bacteria. Values shown on each node of the phylogenetic tree are bootstrap values. A total of 2,000 bootstrap replicates were performed.

The culture dependent approach was the conventional dilution-plating method using a selective mineral medium with oil-vapor as a sole source of carbon and energy for hydrocarbonoclastic bacteria, as well as two conventional media; nutrient agar and marine agar. The crude oil samples were free from any living microorganisms. Obviously, the concentrated toxic constituents especially the polyaromatic hydrocarbons in addition to the harsh physicochemical parameters are responsible for this microbial sterility. Total counts of the gel bacteria on those media were 2.5 × 10^6^, 3.1 × 10^6^ and 3.6 × 10^6^ colony forming units (CFU) g^−1^, respectively. The constituent bacterial isolates were purified and identified by comparing the sequences of their 16S rRNA-genes with those of type strains in the GenBank database. The results of this analysis are summarised in Table 1. The phylogenetic tree in Fig. 4 shows the relationships among bacterial isolates on the three media used. The medium selective for hydrocarbonoclastic bacteria and nutrient agar favored Gammaproteobacteria predominantly affiliated with *Alcanivorax* spp., where as the marine agar favored mainly Alphaproteobacteria affiliated with *Thalassospira australica* and Actinobacteria affiliated with *Kocuria flava* as minor taxa. All these bacterial species had been isolated earlier from the Arabian Gulf by our group and reported as hydrocarbon degraders^27,28^. Experimental data will be presented below showing that the strains isolated from the gels were actually effective hydrocarbonoclastic bacteria.

**Table 1:** Sequencing of hydrocarbonoclastic bacteria isolated from the gels using the culture-dependent method.

Isolate no.	Total bases	Subdivision	Nearest GenBank match	Similarity %	Bases compared	GenBank accession no.
Strain 1	496	Gammaproteobacteria	*Alcanivorax venustensis*	100	496/496	MF158171
Strain 2	514	Gammaproteobacteria	*Alcanivorax jadensis*	99	517/518	MF158172
Strain 3	525	Gammaproteobacteria	*Alcanivorax borkumensis*	100	525/525	MF158173
Strain 4	499	Alphaproteobacteria	*Thalassospira australica*	100	499/499	MF158174
Strain 5	508	Actinobacteria	*Kocuria flava*	100	508/508	MF158175
Strain 6	523	Alphaproteobacteria	*Blastomonas natatoria*	99	525/526	MF158176

**Figure 5:**
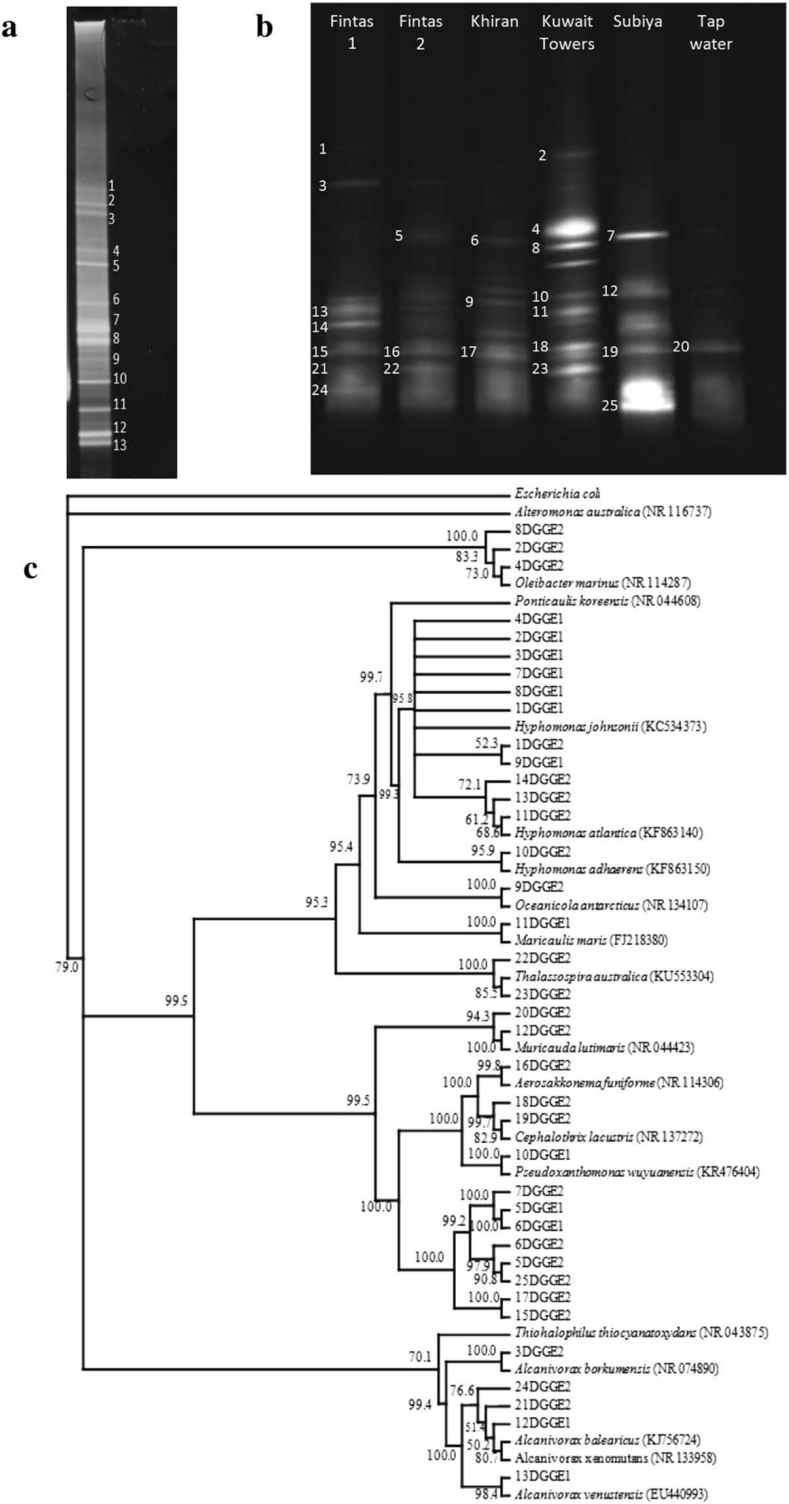
Culture-independent analysis of bacterial communities associated with established oil-gels. (**a**) DGGE Profile of 16S rRNA-gene amplicons in total genomic DNA extract of the 4 months old gel established in Fintas seawater. (**b**) DGGE Profiles of 16S rRNA-gene amplicons in total genomic DNA extracted from gels established for 40 days in water samples from different sites. (**c**) Phylogenetic tree based on 16S rRNA-gene partial sequences, showing the phylogeny of bacterial bands from both DGGE gels.

Bacterial communities associated with established gels were also analyzed by a culture-independent approach. Total genomic DNA was extracted from the gels and the 16S rRNA-genes in the extracts were amplified and resolved by denaturing gradient gel electrophoresis (DGGE). The resolved bands were amplified and sequenced, and the sequences were compared with those of type strains in the GenBank database. The DGGE profile in Fig. 5a shows the 16S rRNA-gene amplicons from total genomic DNA of a 4 month oil-gel in the Fintas seawater. The identities of the resolved bands are listed in Table 2. Nine of the 13 bands were affiliated with *Hyphomonas johnsonii*, 2 with *Alcanivorax* spp., 1 with *Pseudoxanthomonas wuyuanensis* and 1 with *Maricaulis maris*. To recall, our light micrographs (Fig. 3b,c) revealed prosthetic and stalked bacteria. *Hyphomonas* identified by the culture independent analysis is a typical prosthecate, budding bacterium common in marine habitats^29^. *Maricaulis maris* belongs to the *Caulobacter* family common in low nutrient aquatic habitats and biofilms^30–32^. Reportedly, this species produces the longest and thickest stalk of the known *Maricaulis* spp. While *Pseudoxanthomonas* and *Alcanivorax* were frequently recorded in the literature as hydrocarbonoclastic bacteria^27,33^, nothing is known so far, about this activity among *Hyphomonas* spp. and *Maricaulis* spp.

**Table 2:** Sequencing of amplicon bands in Fig. 5a.

Band no.	Nearest GenBank match	Band name	GenBank accession no.
1	*Hyphomonas johnsonii*	1DGGE1	MF093578
2	*Hyphomonas johnsonii*	2DGGE1	MF093579
3	*Hyphomonas johnsonii*	3DGGE1	MF093580
4	*Hyphomonas johnsonii*	4DGGE1	MF093581
5	*Hyphomonas johnsonii*	5DGGE1	MF093582
6	*Hyphomonas johnsonii*	6DGGE1	MF093583
7	*Hyphomonas johnsonii*	7DGGE1	MF093584
8	*Hyphomonas johnsonii*	8DGGE1	MF093585
9	*Hyphomonas johnsonii*	9DGGE1	MF093586
10	*Pseudoxanthomonas wuyuanensis*	10DGGE1	MF093587
11	*Maricaulis maris*	11DGGE1	MF093588
12	*Alcanivorax balearicus*	12DGGE1	MF093589
13	*Alcanivorax venustensis*	13DGGE1	MF093590

The DGGE profiles in Fig. 5b show the 16S rRNA-gene amplicons from the total genomic DNA of 40 days old oil-gel samples established using the Fintas, Khiran, Kuwait Towers, Subiya and tap-water samples. The identities of the bands are listed in Table 3. In addition to various *Hyphomonas* spp. and *Alcanivorax* spp., additional species belonging to the genera; *Oleibacter, Oceanicola, Alteromonas, Ponticaulis, Muricauda, Aerosakkonema, Cephalothrix, Thalassospira and Thiohalophilus* were harbored by the various gel samples. Interestingly, many of the identified genera, viz. *Alcanivorax, Oleibacter* and *Thalassospira* belong to the group of the so-called obligate hydrocarbonoclastic bacteria (OHB) reported to be major degraders of spilled oil in the marine environment^33^. Also *Alteromonas* ^34^ has been reported as hydrocarbonoclastic. The list in Table 3 probably comprises other hydrocarbonoclastic taxa whose hydrocarbon utilization potential has not yet been studied.

**Table 3:** Sequencing of amplicon bands in Fig. 5b.

Band no.	Nearest GenBank match	Band name	GenBank accession no.
1	*Hyphomonas johnsonii*	1DGGE2	MF093591
2	*Oleibacter marinus*	2DGGE2	MF093592
3	*Alcanivorax borkumensis*	3DGGE2	MF093593
4	*Oleibacter marinus*	4DGGE2	MF093594
5	*Oleibacter marinus*	5DGGE2	MF093595
6	*Alteromonas australica*	6DGGE2	MF093596
7	*Oleibacter marinus*	7DGGE2	MF093597
8	*Ponticaulis koreensis*	8DGGE2	MF093598
9	*Oceanicola antarcticus*	9DGGE2	MF093599
10	*Hyphomonas adhaerens*	10DGGE2	MF093600
11	*Hyphomonas atlantica*	11DGGE2	MF093601
12	*Muricauda lutimaris*	12DGGE2	MF093602
13	*Hyphomonas atlantica*	13DGGE2	MF093603
14	*Hyphomonas atlantica*	14DGGE2	MF093604
15	*Aerosakkonema funiforme*	15DGGE2	MF093605
16	*Aerosakkonema funiforme*	16DGGE2	MF093606
17	*Aerosakkonema funiforme*	17DGGE2	MF093607
18	*Cephalothrix lacustris*	18DGGE2	MF093608
19	*Cephalothrix lacustris*	19DGGE2	MF093609
20	*Cephalothrix lacustris*	20DGGE2	MF093610
21	*Alcanivorax xenomutans*	21DGGE2	MF093611
22	*Thalassospira australica*	22DGGE2	MF093612
23	*Thalassospira australica*	23DGGE2	MF093613
24	*Alcanivorax xenomutans*	24DGGE2	MF093614
25	*Thiohalophilus thiocyanatoxydans*	25DGGE2	MF093615

### Removal of hydrocarbons in established oil-gels

**Figure 6:**
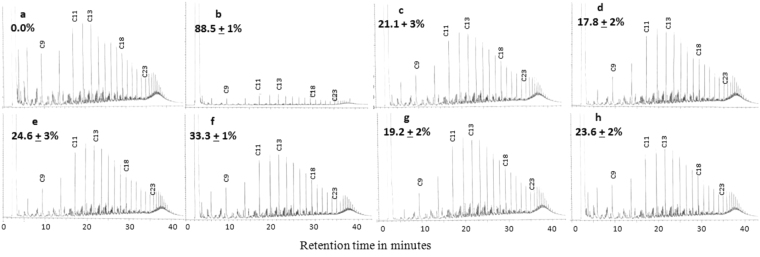
GLC Profiles of hydrocarbons recovered from an established gel and from oil-containing batch cultures supporting hydrocarbonoclastic bacteria isolated from the gels. (**a**) Crude oil at time zero. (**b**) Oil recovered from the gel established after 4 months in Fintas seawater. (**c**) Oil recovered from *Alcanivorax venustensis* culture batch. (**d**) Oil recovered from *Alcanivorax jadensis* culture batch. (**e**) Oil recovered from *Alcanivorax borkumensis* culture batch. (**f**) Oil recovered from *Thalassospira australica* culture batch. (**g**) Oil recovered from *Kocuria flava* culture batch. (**h**) Oil recovered from *Blastomonas natatoria* culture. Values on the individual profiles are those of the hydrocarbon consumption values, they were means of 3 replicates ± standard deviation values.

The GLC-profiles of constituent hydrocarbons of the oil added at time zero (oil used for gelation) and in 40 days old gels (Fig. 6a,b, respectively) show that those hydrocarbons have been effectively removed. Quantitation in terms of total peak area decrease revealed that 88.5% of the constituent hydrocarbons were biodegraded within 40 days.

The other profiles (Fig. 6c–h) show that individual microbial isolates from the established oil-gels in batch cultures removed between 17.8 and 33.3% of crude oil after 12 days of incubation. Most effective was *Thalassospira australica* with 33.3% oil removal, and the least effective was *Alcanivorax jadensis* with 17.8% oil removal. These results confirm that the gelators naturally produced are quite promising, not only as tools for mechanical oil removal, but also because they harbor rich microbial consortia capable of mineralizing the physically entrapped oil. This novel result is valuable for suggesting new biotechnologies for removal of oil spilled in aqueous environment.

## Discussion

There is increasing focus on basic research in the field of oil-gelators as a means of mechanical remediation of oil spilled in aquatic environments^8,9^. However, this research is on one hand dealing with artificially synthesized gelators, and on the other is limited to the chemistry and physical chemistry of those gelators.

The novelty of the current study lies in that it represents the first attempt of considering the oil gelation phenomenon from the microbiological point of view. This study is also the first in the available literature that deals with natural gelators. Although the major objectives did not propose to determine the chemical composition of such natural gelators, the results excluded the possibility that they might be amino acid-^15^, peptide-^17^ or carbohydrate-^18^ based, as most of the synthetic gelators reported in the literature are. On the other hand, the results provide some tentative evidence that our natural gelators could be cholesteryl derivatives, like the few synthetic gelators reported in the literature^19,20^. However, this evidence was based solely on the TLC-analysis and is thus, inadequate. More conclusive evidence for the chemical composition of this gelator is still needed.

Irrespective of the chemical identity of the natural gelator, it is biotechnologically interesting that inoculation of whole-oil mixtures with pieces of already established gels enhanced the process. As already mentioned, chemical gelators in the small inocula might have acted as “starters” in the self-assembly of the nanofibrillar structures. This speculation still needs experimental evidence. The results of this study showed that crude oil samples, especially heavy crudes comprise natural oil-gelators, which irrespective of their chemical composition when vigorously shaken with water frequently lead to spontaneous oil-gelation at moderate temperatures leaving the water perfectly clear. Earlier workers on synthetic oil gelators also showed that mechanical agitation was among the stimuli effective in gelation^7^. Gelators have been reported to create supermolecular networks by self assembly which entrap the gelled materials^1,2^. Within this context the SEM of our gels revealed nanofibrillar structures, which are probably involved in the oil entrapment. Interestingly, the oil gels proved to harbor rich bacterial communities, which comprised prosthatic and stalked bacteria as well as bacterial species that were effective in oil degradation in batch cultures. In view of the experimentally revealed microbiological sterility of crude oil (see results) and the similarity of the bacterial composition of the studied gels to that of the Arabian-Gulf water^27^, it may be concluded that the constituent bacterial species in the gels had their origin in the seawater used. Confirming this assumption is that the gels created using autoclaved seawater were free from bacteria (Results). Established oil-gels naturally lose their gelatinized oil after some time via the activities of their inhabitant hydrocarbonoclastic bacteria. The occurrence of such bacterial communities in the gels has been revealed using culture-dependent and culture-independent microbiological methods. Both approaches confirmed the frequent occurrence of typical marine hydrocarbon utilizing bacteria in the gel, e.g. *Alcanivorax spp*., *Thalassospira spp*., *Kocuria sp*., *Pseudoxanthomonas sp*., *Oleibacter sp*. and many others. The gels also harbored a rich community of appendage bacteria viz *hyphomicrobium spp*. and *Maricaulis sp*., know to be carbooligotrophic and whose hydrocarbonoclastic potential is probable. This novel finding represents information quite valuable when suggesting remediation technologies for oil spilled in aquatic environments. The approach would depend on oil entrapment from water in the first step, followed by subsequent biodegradation of this entrapped oil via the activities of the microbial communities naturally harbored by the oil-gels.

## Methods

### Seawater sampling

Fresh seawater samples were collected in sterile conical flasks. They were transported to the laboratory and started to be processed the same day. In some experiments, water samples were stored at room temperature for weeks, before they were used. Environmental parameters in the sampling sites were recorded using a water quality checker (Horiba, Japan).

### Oil-gelation experiments

Three parallel treatments were prepared throughout. For gelation, 100 ml aliquots of seawater were dispersed in sterile flasks. Each flask was provided with 1 g Kuwaiti crude oil (light and heavy). Flasks were incubated at 30 °C on an electrical shaker, 200 rpm, and parallel flasks were left stationary at 30 °C. The mixtures were examined daily for gelation. Gels were separated from the seawater samples by using conventional metal sieves, about 1 mm mesh.

### Chemical analysis of established gels

Established gels were obtained, and were washed with sterile seawater, ready for chemical analysis.

Elemental analysis was done using ICP-OES-Perkin Elmer instrument adopting the USEPA 6010B and USEPA 3050B methods.

Established gel samples, 2 g, were subjected to acid (1 N H_2_SO_4_)- and alkaline (1 N NaOH)- hydrolysis. The hydrolysates were analyzed by thin layer chromatography (TLC) for amino acids and monosaccharides^35^.

Gel samples were also subjected to instrumental analysis using Liquid Chromatography-Mass Spectrometry/Mass Spectrometry (LC-MS/MS) to test them for peptide and polysaccharide residues.

### Analysis of steryl ester derivatives in established gels

Total lipids in the established gels, as well as in other environmental samples e.g. the local fish (*Nemipterus randalli*) were studied. Total lipids were extracted following established procedures^36^ and the lipid classes were resolved Authentic, standard lipid classes were co-chromatographed for identification by TLC. Precoated Silica Gel G plates (MERCK, Germany) were used. The lipid classes, comprising sterols and steryl esters, were resolved by the solvent mixture, hexane, diethylether, acetic acid (85:15:1, by vol). The lipid classes were visualized by spraying the plates with 10% phosphomolybdic acid in ethanol and 50% H_2_SO_4_, followed by heating the plates at 120 °C.

### Microscopic examination

For light microscopy, small oil-gel pieces were macerated in drops of seawater on a clean microscopic slide and examined (bright field). Micrographs were recorded using OLYMPUS DP12 Microscope Digital Camera.

For scanning electron microscopy (SEM), a small piece of the oil-gel was placed on copper tape attached aluminum stub, and allowed to dry overnight under ambient conditions. The sample was sputter-coated with a thin layer of platinum and examined by Leo (Zeiss) Remotely Operationable Variable Pressure Field Emission SEM.

Analysis of hydrocarbonoclastic bacterial communities in established gels. Total hydrocarboclastic bacteria associated with the established gels were analyzed by a culture-dependent method using a mineral medium^21^ with oil vapor as a sole source of carbon and energy. In a recent publication, our group documented the history of this method and its use for counting and analysis of bacterial communities in environmental samples^37^. Aliquots, 0.1 ml, of a gel dilution series in sterile water were spread on the surfaces of the solid medium in plates. Each inoculated plate was inverted over its lid that had been provided with a filter paper impregnated with 2 ml crude oil as a source of oil vapor. The plates were sealed with cello-tape and incubated at 30 °C for 14 d. Three replicate plates were prepared throughout. The numbers of colony forming units (CFU) g^−1^ were counted and mean values calculated. Parallel plates were pooled and, following established procedures similar bacterial strains were differentiated by the morphologies of the colonies (size, texture, color, margin, etc.) and cell characteristics (shape, size, Gram staining, inclusions, motility, etc.). Morphological differences are known to reflect genetic variations. Representative colonies were subcultured and purified. The pure cultures were characterized, their genomic DNA was extracted, and their 16S rRNA-coding genes were amplified. The resulting amplicons were sequenced and the sequences compared with the nearest GenBank sequences. To extract the total genomic DNA, 300 mg of the fresh 48-h biomass was homogenized in 100 µl of PrepMan Ultra Sample Preparation Reagent (Applied Biosystems, USA) and 200 µl molecular water (Sigma, UK). The mixture was incubated in a water bath for 10 min at 100 °C, cooled for 2 min and centrifuged at 14,000 x *g* for 3 min to collect the DNA-containing supernatant. The 16S rRNA-genes were amplified by polymerase chain reaction (PCR). The reaction mixture contained puReTaq Ready-To-Go PCR Beads (Amersham Biosciences, UK), 1 µl (25 ng) of DNA templat and 1 µl each of the universal primer combinations GM5F (50-CCTACGGGAGGCAGCAG-30) and 907 R (50-CCGTCAATTCMTTTGAGTTT-30)^38^. The reaction volume was completed to 25 µl with molecular water. Amplification was done in a Veriti Thermal Cycler (Applied Biosystems, USA) by a touch-down PCR in which the initial denaturation was at 95 °C for 5 min, and the annealing temperature started at 65 °C and decreased by 1 °C every cycle to 55 °C, at which additional 15 cycles were carried out. Denaturation was at 94 °C for 1 min, and primer extension at 72 °C for 1 min. The PCR products were purified using a QIA quick PCR purification kit (Qiagen, USA) in order to remove the Taq polymerase, primers and dNTPs. Partial sequencing of the 16S rRNA-gene was done using a BigDye version Terminator Kit (Applied Biosystems, USA); 20 ng of the DNA template was added to 2 µl of a Big Dye v 3.1 terminator and 2 µl of Big Dye Terminator v 1.1, v 3.1 5X sequencing buffer; l µl of either 907 R or GM5F was added to the mixture, and the final volume was brought up to 10 µl with molecular water. Labeling was completed in a Veriti Thermal Cycler (Applied Biosystems, USA) using one cycle of 96 °C for l min, then 25 cycles of l min at 96 °C, 5 s at 50 °C and 4 min at 60 °C. The pure template DNA samples were processed in a 3130xl genetic analyzer (Applied Biosystems, USA). Sequencing analysis version 5.2 software (Applied Biosystems, USA) was used to analyze the results. Sequences were subjected to basic local alignment search tool analysis with the National Center for Biotechnology Information (NCBI; Bethesda, MD, USA) GenBank database^39^. A phylogenetic tree was constructed using neighbor-joining bootstrap proportions, based on 2000 replicates.

Molecular analysis of total bacterial communities in established gels. The total bacterial communities harbored by the gels were also analyzed by a culture-independent method. Total genomic DNA was extracted from established gel samples and the 16S rDNA-genes therein were amplified using the primer combination named above with adding a GC-clamp (CGCCCGCCGCGCGCGGCGGGCGGGGCGGGGGCACGGGGG) to the GM5F primer^40^. The amplicons were resolved by DGGE using a Dcode Universal Mutation Detection System (Bio-Rad, California, USA). The denaturant concentrations were 45–55%. Electrophoresis was processed with the constant voltage of 50 V at 60 °C for 16 h. Gels were stained with SYBR Green (Invitrogen, USA) in 1X TAE buffer (1:100000) for 30 min, and examined using a Dark Reader transilluminator (Clare Chemical Research, CO, USA). Individual gel bands were stored in 50 µl molecular water (Sigma, UK) at 4 °C overnight to elute the DNA, and the DNA in 1 µl was amplified, purified and sequenced as described above.

Measurement of hydrocarbon consumption. Hydrocarbons in the established gels and in the culture batches were recovered by extraction using three successive aliquots of pentane, 15 ml each, the combined extract was completed to 50 ml using pentane and 1 µl was analyzed by gas liquid chromatography (GLC), using a Chrompack (NJ, USA) CP-9000 instrument equipped with a flame ionization detector, a WCOT fuse silica capillary column and a temperature program, 45–310 °C, raising the temperature 10 °C min^−1^. Nitrogen was the carrier gas; the detector temperature was 250 °C and the injector temperature 150 °C. The hydrocarbon-consumption was calculated in terms of percent total peak area reduction in the GLC profiles based on the peak areas of the crude oil amount added at time zero.
